# Comparison of the effects of rapid maxillary expansion versus Twin Block appliance on mandibular growth in skeletal Class II patients

**DOI:** 10.1186/s12903-020-01344-8

**Published:** 2020-12-01

**Authors:** Jia-Nan Zhang, Si Chen, Cheng-Yi Huang, Chong Zhong, Jing Jin, Feng-Yang Yu, Zan-Zan Zhang, Hai-Ping Lu

**Affiliations:** 1 Center of Orthodontics, Department of Dentistry, Sir Run Run Shaw Hospital, Zhejiang University School of Medicine, 3# Qingchundong Road, Hangzhou, China; 2 Department of Orthodontics, Peking University School and Hospital of Stomatology, 22# Zhongguancun S. Ave., Beijing, China; 3 Center of Orthodontics, Perfect Dental Care, 108# Xintang Road, Hangzhou, China; 4 Department of Orthodontics, College of Stomatology, Zhejiang Chinese Medical University, 548# Binwen Road, Hangzhou, China

**Keywords:** Rapid maxillary expansion, Twin-Block, Mandible, Growth, Class II malocclusion

## Abstract

**Background:**

This is a retrospective study that compares mandibular growth changes in skeletal Class II patients treated by rapid maxillary expansion (RME) and following fixed appliance with those patients treated by Twin-Block (TB) and following fixed appliance.

**Methods:**

Fourteen patients treated by RME and following fixed appliance were included into the RME group. Fifteen patients treated by Twin-Block and following fixed appliance were included into the TB group. Lateral cephalometric radiographs taken before treatment and immediately after fixed appliance treatment were used to evaluate mandibular growth effects.

**Results:**

The starting forms of the patients in the two groups were examined to be of good comparability. The mandibular length increased significantly in both groups as measured by Co-Gn, Go-Gn and Ar-Gn, but the TB group didn’t show more mandibular growth than the RME group (*P* > 0.05). Skeletal changes of the mandible in vertical dimension were different in the two groups. The change in FMA was 0.35° in the RME group, while the change was 2.65° in the TB group (*P* < 0.001). The change in LAFH was 5.14 mm in the RME group, significantly smaller than the change of 10.19 mm in the TB group (*P* < 0.001).

**Conclusion:**

The investigated Phase I treatment with RME followed by Phase II treatment of fixed appliance achieved the same increases in sagittal mandibular growth and facial profile improvements as the Twin-Block therapy. The treatment with RME followed by fixed appliance was better for vertical control, while the treatment with Twin-Block followed by fixed appliance significantly increased the mandibular plane angle.

## Background

Class II malocclusion, a common problem occurring in approximately one-third of the population, is often accompanied by mandibular retrusion [1]. Therefore, the successful management of mandible growth is key to Class II malocclusion treatment.

Many orthopedic treatment methods have been introduced to advance mandibular growth. As a method that directly facilitates sagittal growth of the mandible, Twin-Block (TB) is a classic appliance for skeletal Class II correction [2]. TB promotes mandibular growth by directly leading the mandible to a forward position to enable condyle remodeling at the new position; however, side effects of TB include downward and backward rotation of the mandible, increase in lower facial height, and labial tipping of the lower anterior teeth [3].

The transverse maxillary dimension also affects the sagittal position of the mandible. Maxillary transverse deficiency is a common characteristic of skeletal Class II malocclusion [4]. McNamara [5] discovered that during palatal expansion, a spontaneous sagittal correction occurred in skeletal Class II patients with slight or moderate mandibular retrusion. Later, many studies also demonstrated a strong linear correlation between boned rapid maxillary expansion (RME) therapy and forward growth of the mandible [6, 7].

The key point of achieving anteroposterior growth of the mandible by RME therapy lies in the space acquired by separating the mid-palatal suture. Once the mid-palatal suture is expanded and space is created, the blocked occlusion between the upper and lower dentition is relieved, enabling the mandible to naturally grow in three dimensions [7]. As Vargervik [8] demonstrated, the correction of Class II malocclusion requires an increase in the maxillary molar width, by approximately 2 mm for a unilateral Class II molar relationship and 4 mm for a bilateral Class II molar relationship.

Whether the maxilla or the mandible is the key for skeletal Class II correction remains unsettled. Additionally, whether RME-induced mandible growth or TB-induced mandible reposition/growth is more stable for long-term correction remains to be answered. To the best of our knowledge, although a few studies have examined mandibular growth in the sagittal and vertical dimensions immediately after RME therapy or Twin-Block therapy [9, 10], no study has compared the final effects of the two therapies on mandibular growth after the completion of comprehensive orthodontic treatment. The objective of this study is to compare the effects of RME versus TB on mandibular growth in orthopedic-orthodontic combined treatment.

## Methods

This retrospective study was approved by the Research Ethics Committee of Sir Run Run Shaw Hospital, Zhejiang University School of Medicine. Sample size was calculated considering a mean difference of 4.5 mm between groups for the amount of total mandibular length (Co-Gn), contemplated as the primary outcome, with a previously reported standard deviation about 2.9 mm [7, 10], using 80% test power, at 5% alpha level. Then, a minimum of 9 patients was necessary in each group.

The inclusion criteria were as follows:

1. Patients of 8–14 years of age with mixed dentition;
2. Patients with a skeletal Class II pattern, ANB 5°-8°, SNB < 78°;
3. Growing patients with a cervical vertebral maturation (CVM) stage between 2–4 period;
4. Two-phase treatment consisting of Phase I RME or Twin-Block therapy, followed by Phase II fixed appliance treatment with four 1^st^ premolar extractions;
5. Patients with accurate and complete records, containing two good-quality cephalometric radiographs, taken before treatment (T1) and immediately after fixed appliance treatment (T2).

The exclusion criteria were as follows:

1. Patients with an extremely increased vertical growth tendency, reflected by mandibular plane angle (FMA) > 36°;
2. Patients with congenitally missing teeth;
3. Patients with visible posterior crossbite.

A total of 29 patients were selected according to the inclusion and exclusion criteria. Fourteen patients were included in the RME group, including 4 boys and 10 girls. And the remaining fifteen patients were included in the TB group, including 9 boys and 6 girls.

The matching analysis between the two groups was based on the characteristics of chronologic age, stage of CVM, cephalometric measurements and treatment duration.

### Treatment protocols

In the RME group, rapid maxillary expansion was used for phase I treatment. The bonded RME was composed of an expansion screw in the center of the palate and the acrylic splint covering the posterior teeth. The RME was activated via two turns per day (0.25 mm per turn) for a period of 2 weeks. Then the bonded RME was maintained in place at a passive state for approximately 6 months to allow for stabilization. Immediately after RME debonding, fixed appliances were placed. During phase II treatment, four 1st premolars were extracted, and mini-screws were placed in the maxilla for maximum anchorage.

In the TB group, Twin-Block was used for phase I treatment. The posterior bite blocks were ground every 4–6 weeks, until the posterior teeth established occlusion. Then, the Twin-Block was left in the mouth for 3 months to maintain stabilization. Immediately after Twin-Block removal, fixed appliances were placed. During phase II treatment, four 1st premolars were extracted, and mini-screws were placed in the maxilla for maximum anchorage.

After completion of the phase II treatment, all the patients in both groups were instructed to wear the circumferential Hawley retainer for 24 h a day.

### Cephalometric analysis

T1 and T2 cephalometric radiographs were uploaded into Dolphin software (Version 11.9, Dolphin Digital Imaging, Chatsworth, Calif, USA). Two investigators simultaneously traced the anatomic contours and located landmarks. Any disagreements about landmark location were resolved by retracing the anatomic contours until the two investigators achieved the same point. Location of the landmarks and calculation of the measurement items were repeated three times. The consistency of the measurements was tested.

**Fig. 1:**
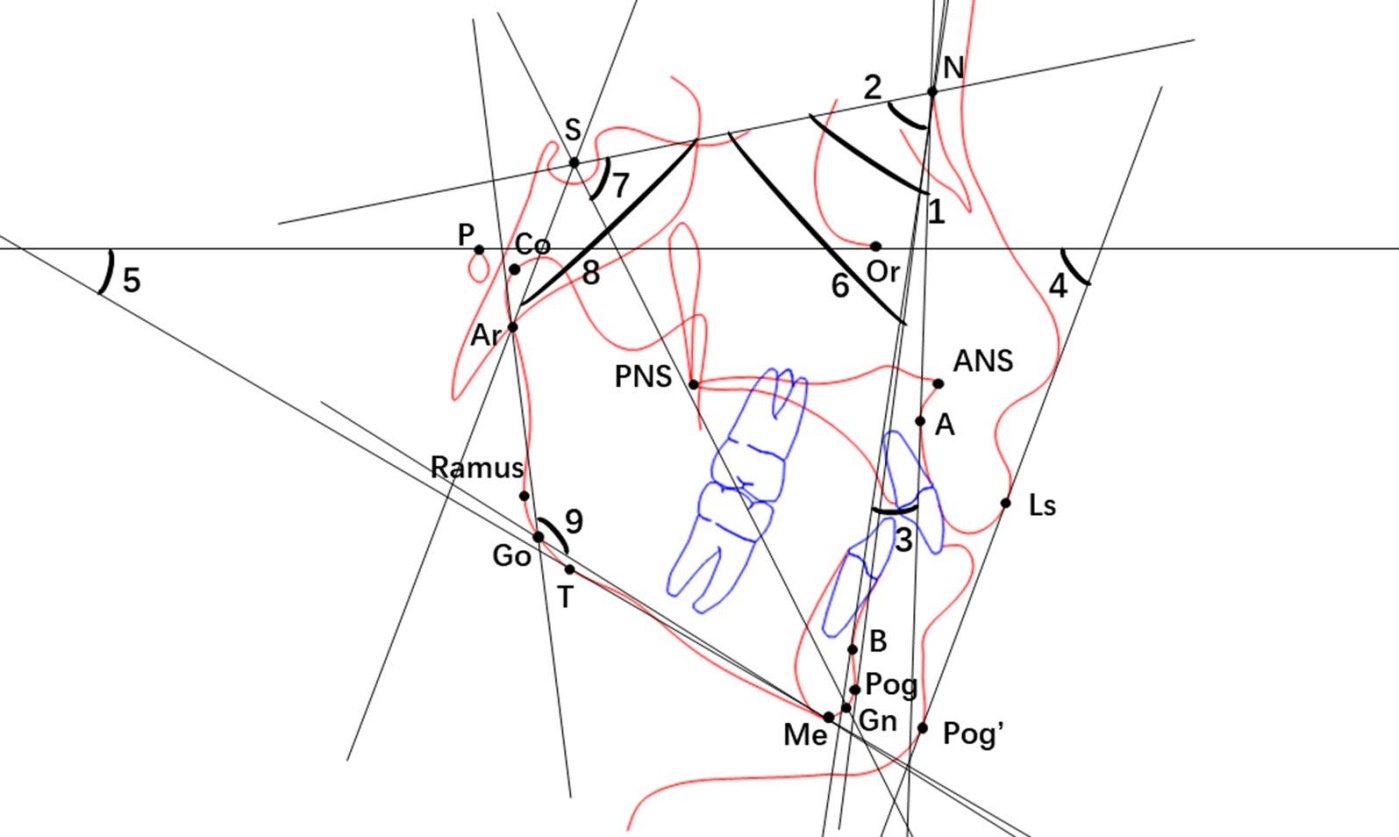
Cephalometric tracing illustrating 9 angular measurements: 1, SNA; 2, SNB; 3, ANB; 4, Z Angle; 5, FMA; 6, SNPog; 7, NSGn; 8, NSAr; 9, ArGoMe

**Fig. 2:**
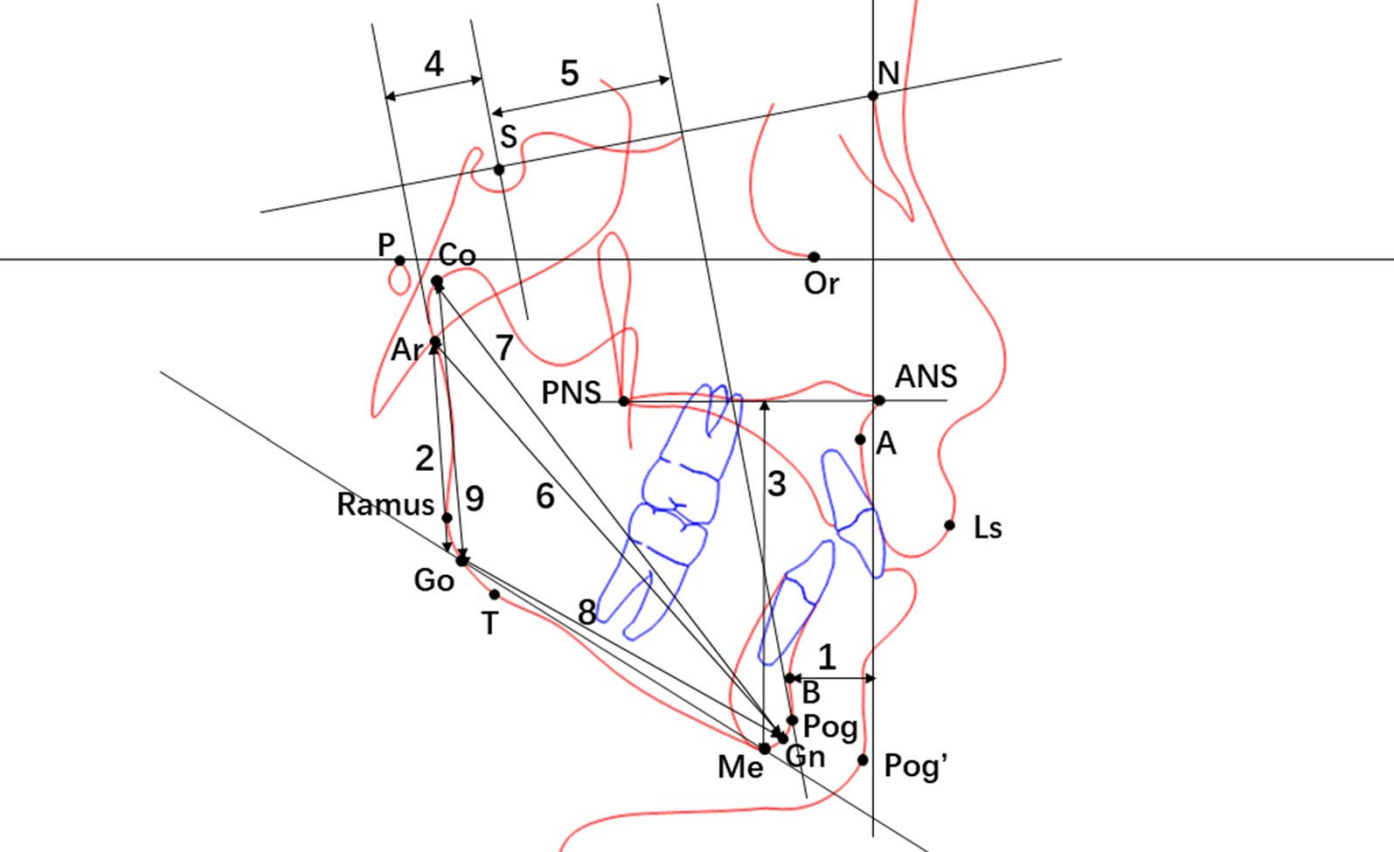
Cephalometric tracing illustrating 9 linear measurements: 1, Pog-N|FH; 2, PFH; 3, LAFH; 4, SE; 5, SL; 6, Ar-Gn; 7, Co-Gn; 8, Go-Gn; 9, Co-Go

The analyzed angular and linear cephalometric measurements at T1 and T2 within each group included SNA, SNB, ANB, Z Angle, FMA (mandibular plane to FH), SNPog (facial plane to SN), NSGn (Y-Axis), NSAr (Sella Angle), ArGoMe (Gonial Angle), Pog-N|FH (pogonion to nasion perpendicular), PFH (posterior facial height), LAFH (lower anterior facial height), PFH/LAFH, SE (Steiner Analysis), SL (Steiner Analysis), Ar-Gn (effective mandibular length), Co-Gn (total mandibular length), Go-Gn (mandibular body length), and Co-Go (mandibular ramus length). These measurements are displayed in Figs. 1 and 2.

### Statistical analysis

The data were analyzed by IBM SPSS Statistics 20.0 (IBM Corp., Armonk, NY, USA). The consistency of the three cephalometric item calculations was tested by the intraclass correlation (ICC). The average measurements of chronologic age, stage of CVM, treatment intervals, and all cephalometric items of the two groups at T1 and T2 were used for analysis.

Exploratory chi-square statistical tests were performed to examine the normal distribution of chronologic age, CVM stage, treatment intervals, and all the cephalometric measurements in the two groups.

An independent-samples T test was performed to compare chronologic age and CVM stage at T1, and treatment intervals between the two groups. Additionally, an independent-samples T test was conducted to compare cephalometric measurements between the two groups at T1 and T2. A paired-samples T test was performed to analyze the changes from T1 to T2 within each group. Finally, the changes in the cephalometric measurements from T1 to T2 between the two groups were compared using an independent-sample T test. Statistical significance was determined at the levels of *P* < 0.05, *P* < 0.01 and *P* < 0.001.

## Results

The ICC coefficients of cephalometric measurements were higher than 0.819, with the 95% confidence interval ranged from 0.623 to 0.996 among the measurements. The results of the exploratory chi-square statistical tests supported the hypothesis of normality of distribution for the examined parameters.

The general information of the patients in the two groups is summarized in Table 1. The statistical results showed that neither the chronologic age nor the CVM stage before treatment significantly differed between the two groups (*P* > 0.05). During the phase I period, the average treatment duration in the RME group was shorter than that in the TB group (*P* < 0.05). No significant difference in the treatment time between the two groups in phase II was observed (*P* > 0.05).

**Table 1:** The general information of the patients in the two groups

Basic characteristics	RME group	TB group	*P*
Chronologic age (year)	10.94 ± 1.40	10.41 ± 1.06	0.258
CVM Stage	3.79 ± 0.58	3.60 ± 0.51	0.365
Treatment interval of phase I (month)	6.86 ± 1.88	10.80 ± 5.35	0.015*
Treatment interval of phase II (month)	38.86 ± 12.17	35.87 ± 10.99	0.493

Data are presented as mean ± SD; *P* < 0.05 = significant; *P < 0.05

The descriptive data and comparative statistics of cephalometric measurements in the two groups are presented in Table 2. When the cephalometric measurements at T1 were evaluated, no significant difference was detected between the two groups (*P* > 0.05).

**Table 2:** The descriptive data and comparative statistics of cephalometric measurements in the two groups

Cephalometric Measurements	RME Group				TB Group				P_1_	P_2_	P_c_
	T1	T2	Change	P_1-2_	T1	T2	Change	P_1-2_			
Regular items											
SNA (°)	80.73 ± 2.75	79.91 ± 2.35	− 0.82 ± 1.37	0.042*	80.82 ± 2.73	79.73 ± 3.82	− 1.09 ± 2.16	0.072	0.929	0.885	0.698
SNB (°)	74.29 ± 2.34	75.90 ± 2.35	1.61 ± 1.19	0.000^‡^	74.15 ± 2.44	75.61 ± 3.52	1.47 ± 1.73	0.005^†^	0.877	0.800	0.792
ANB (°)	6.44 ± 0.96	4.01 ± 1.24	− 2.44 ± 1.19	0.000^‡^	6.67 ± 0.81	4.12 ± 1.70	− 2.55 ± 1.56	0.000^‡^	0.489	0.840	0.822
Z angle (°)	55.60 ± 5.80	67.86 ± 5.84	12.26 ± 6.17	0.000^‡^	54.51 ± 5.20	66.36 ± 6.48	11.85 ± 5.83	0.000^‡^	0.599	0.520	0.855
FMA (°)	28.05 ± 3.70	28.40 ± 3.44	0.35 ± 1.57	0.419	28.67 ± 4.10	31.31 ± 3.95	2.65 ± 1.05	0.000^‡^	0.675	0.044*	0.000^‡^
Mandible to cranio base											
SNPog (°)	74.70 ± 2.45	76.61 ± 2.40	1.91 ± 1.34	0.000^‡^	73.83 ± 2.66	76.37 ± 3.42	2.55 ± 1.43	0.000^‡^	0.366	0.829	0.232
NSGn (°)	74.49 ± 2.48	73.81 ± 2.62	− 0.67 ± 1.26	0.067	72.87 ± 2.74	72.53 ± 3.40	− 0.34 ± 1.54	0.405	0.109	0.269	0.542
NSAr (°)	121.96 ± 3.83	120.03 ± 4.29	− 1.94 ± 2.07	0.004^†^	124.19 ± 3.52	123.93 ± 3.06	− 0.27 ± 1.75	0.566	0.114	0.009^†^	0.026*
Pog-N|FH (mm)	10.92 ± 4.53	9.59 ± 7.10	− 1.34 ± 4.56	0.293	14.12 ± 4.25	12.35 ± 6.78	− 1.77 ± 6.00	0.273	0.060	0.292	0.830
Skeletal mandibular items											
PFH (mm)	42.54 ± 2.65	47.28 ± 5.09	4.74 ± 3.62	0.000^‡^	42.22 ± 3.07	48.05 ± 4.50	5.83 ± 3.47	0.000^‡^	0.770	0.670	0.418
LAFH (mm)	57.24 ± 3.74	62.38 ± 3.76	5.14 ± 2.88	0.000^‡^	55.99 ± 3.18	66.19 ± 4.52	10.19 ± 3.13	0.000^‡^	0.343	0.021*	0.000^‡^
PFH/LAFH (%)	74.54 ± 5.74	75.84 ± 7.39	1.30 ± 5.02	0.352	75.55 ± 5.80	72.70 ± 6.07	− 2.84 ± 3.93	0.014*	0.643	0.221	0.020*
SE (mm)	16.75 ± 2.28	17.85 ± 2.68	1.10 ± 1.33	0.009^†^	17.85 ± 2.54	20.07 ± 2.60	2.23 ± 1.56	0.000^‡^	0.233	0.032*	0.046*
SL (mm)	33.32 ± 5.05	37.11 ± 5.26	3.79 ± 3.48	0.001^†^	33.63 ± 4.36	38.35 ± 6.45	4.71 ± 3.10	0.000^‡^	0.860	0.577	0.454
Ar-Gn (mm)	94.72 ± 6.00	104.54 ± 8.02	9.81 ± 5.47	0.000^‡^	91.08 ± 4.04	102.59 ± 5.74	11.51 ± 4.85	0.000^‡^	0.064	0.457	0.383
Co-Gn (mm)	100.04 ± 6.36	112.01 ± 8.39	11.97 ± 5.58	0.000^‡^	96.66 ± 5.99	109.41 ± 7.48	12.75 ± 6.21	0.000^‡^	0.152	0.385	0.725
Go-Gn (mm)	68.44 ± 4.39	74.31 ± 5.63	5.88 ± 3.42	0.000^‡^	66.37 ± 3.60	73.73 ± 4.98	7.37 ± 4.13	0.000^‡^	0.175	0.770	0.302
Co-Go (mm)	53.94 ± 3.58	60.23 ± 5.48	6.29 ± 3.86	0.000^‡^	51.82 ± 3.29	59.89 ± 6.57	8.07 ± 4.59	0.000^‡^	0.108	0.883	0.268
ArGoMe (°)	122.45 ± 3.78	123.86 ± 4.97	1.41 ± 2.81	0.083	119.55 ± 5.67	121.43 ± 6.01	1.88 ± 2.24	0.006^†^	0.120	0.248	0.625

Data are presented as mean ± SD; T1: before treatment; T2: completion of phase II treatment; P_1-2_: the difference of cephalometric measurements before and after treatment in each group; P_1_: the difference of cephalometric measurements between the RME group and the TB group at T1; P_2_: the difference of cephalometric measurements between the RME group and the TB group at T2; P_c_: the difference of changes of cephalometric measurements from T1 to T2 between the RME group and the TB group; *P* < 0.05, *P* < 0.01 and *P* < 0.001 = significant; *P < 0.05; ^†^*P* < 0.01; ^‡^*P* < 0.001

The compliance of the patients included in this study were good and they wore the Hawley retainers as instructed. The facial profile and occlusal relationship remained stable in the one-year follow-up.

### Analysis of treatment effects

From T1 to T2, significant changes in twelve measurements (SNB, ANB, Z Angle, SNPog, PFH, LAFH, SE, SL, Ar-Gn, Co-Gn, Go-Gn, and Co-Go) were observed in both groups (*P* < 0.05). No significant change in NSGn and Pog-N|FH was observed in both groups (*P* > 0.05). SNA and NSAr significantly changed after treatment in the RME group but not in the TB group (*P* > 0.05). FMA, PFH/LAFH and ArGoMe significantly changed after treatment in the TB group but not in the RME group (*P* > 0.05).

Four measurements significantly differed between the two groups at T2. FMA in the TB group was statistically larger than that in the RME group (*P* < 0.05); NSAr was nearly 4° smaller in the RME group than that in the TB group (*P* < 0.01); LAFH in the TB group was much larger than that in the RME group (*P* < 0.05); and SE was smaller in the RME group (17.85 mm) than that in the TB group (20.07 mm) (*P* < 0.05).

### Comparation of the changes

Table 2 summarizes the means, standard deviations and P values of the changes in the cephalometric measurements from T1 to T2 in the two groups. Only the changes in FMA, LAFH, PFH/LAFH, NSAr and SE significantly differed between the two groups.

The change in FMA was approximately 0.35° in the RME group and nearly 2.65° in the TB group (*P* < 0.001). The change in LAFH in the RME group was 5.14 mm, which is significantly smaller than the change in the TB group of approximately 10.19 mm (*P* < 0.001). Additionally, the change in PFH/LAFH in the RME group was 1.30%, which is significantly larger than the change in the TB group of -2.84% (*P* < 0.05).

The change in NSAr was -1.94° in the RME group, which was larger than the change of approximately − 0.27° in the TB group (*P* < 0.05). The change in SE in the RME group was 1.10 mm, which was much smaller than the change of approximately 2.23 mm in the TB group (*P* < 0.05).

## Discussion

RME has been suggested by McNamara [11] as a method to relieve mandibular retrusion in skeletal Class II patients. Twin-Block has been commonly used for phase I treatment in skeletal Class II patients; however, previous studies have only compared the effects of the two therapies on mandibular growth after phase I treatment. No study has compared the final effects of the two therapies on mandibular growth after phase II fixed appliance treatment. Therefore, this study enrolled patients who had completed 2-phase treatment and compared the final results, which were clinically more important.

The physiological age and CVM stage were well matched between the two groups before treatment. No differences in the cephalometric measurement between the two groups at T1 were found. Since the two groups were both treated with four 1^st^ premolar extraction and mini-screws maximum maxillary anchorage for Phase II treatment, the long-term effects of different Phase I orthopedic therapies were responsible for the differences between the final results of the two groups.

**Fig. 3:**
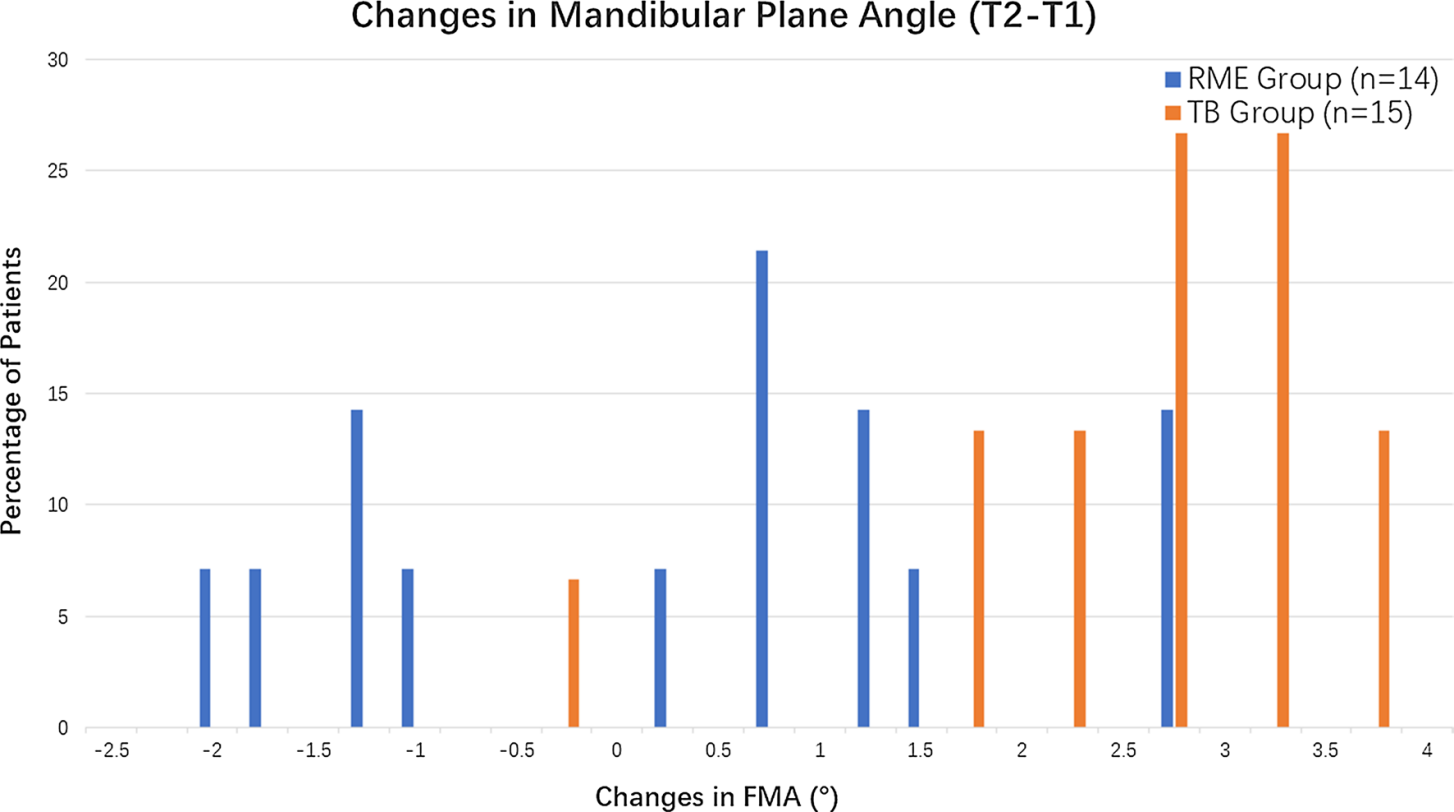
The prevalence rates of patients with different amounts of change in the mandibular plane angle in RME group and TB group

In our research, skeletal changes in the mandible in the vertical dimension were analyzed by angular measurements, including FMA, and liner measurements, such as PFH, LAFH, PFH/LAFH and Co-Go. Although the results indicated an overall increase in the vertical cephalometric measurements after treatment in both groups, a significant difference in the vertical relationship between the two groups was noted. For example, the mean skeletal changes in the mandible in the vertical dimension were much smaller in the RME group than those in the TB group, as reflected by the changes in FMA (Fig. 3). The RME group showed only a slight increase of approximately 0.35° in FMA, suggesting that the vertical dimension was well controlled and that RME therapy might not cause clockwise rotation of the mandible. However, the change in FMA reached 2.65° in the TB group, clearly indicating that Twin-Block therapy might cause clockwise rotation of the mandible.

**Fig. 4:**
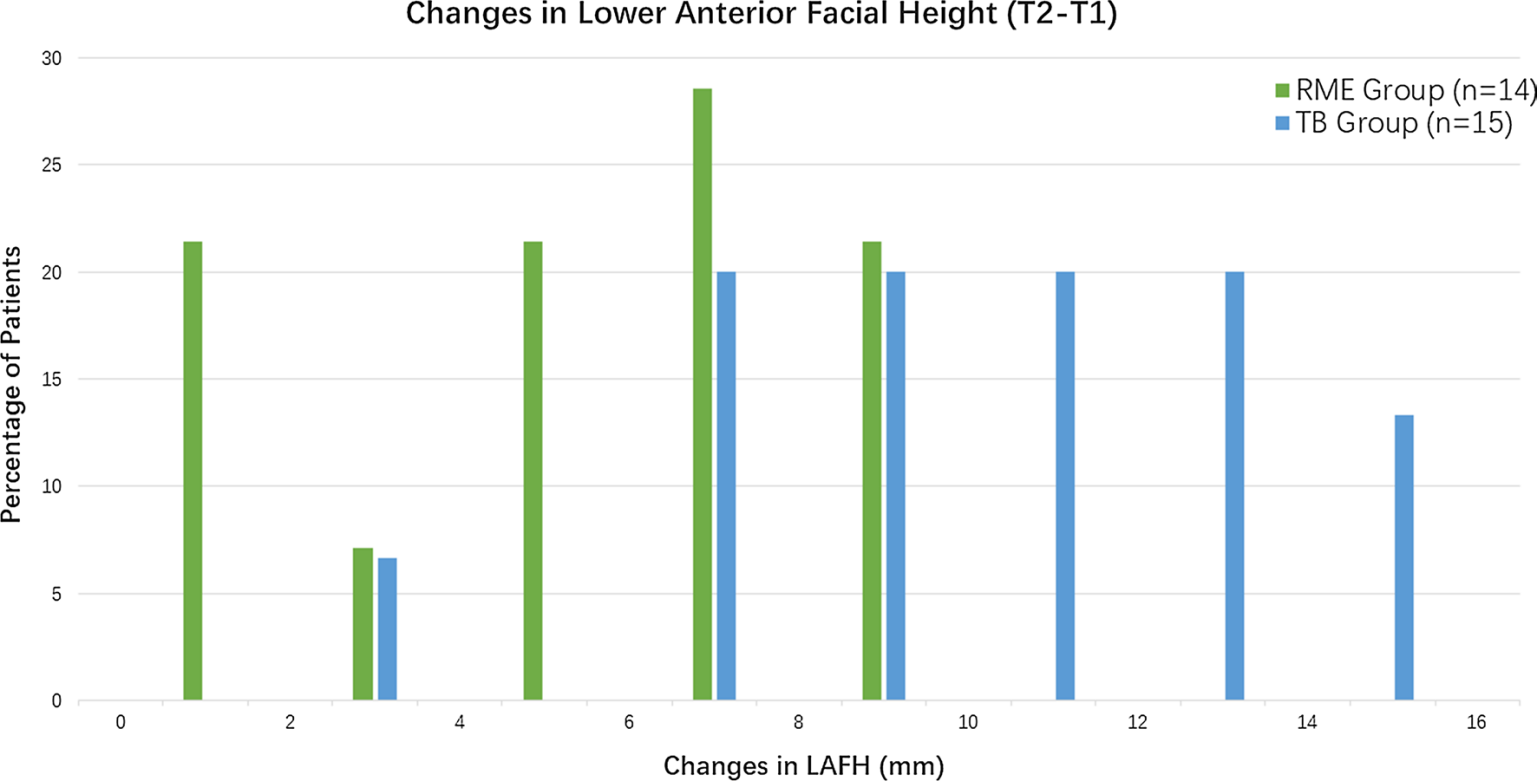
The prevalence rates of patients with different amounts of change in the lower anterior facial height in RME group and TB group

The difference in the FMA change between the two groups could be confirmed by the increase in LAFH, which was 5.12 mm in the RME group and 10.19 mm in the TB group (Fig. 4). In addition, the increase in PFH was similar between the two groups. As a result, the difference in the vertical changes between the two groups revealed an increased tendency of mandibular clockwise rotation following TB therapy. This observation had also been noted by Mills and McCulloch [12]. Moreover, Conroy et al. [13] reported a slight increase in the mandibular plane angle and a slight backward, downward rotation of the mandible after RME treatment, which were consistent with our results.

To evaluate skeletal changes of the mandible in the sagittal dimension, relative angular measurements such as SNB, NSAr, SNPog, and NSGn, and liner measurements such as Pog-N|FH, Ar-Gn, Co-Gn, Go-Gn, SE, and SL, were analyzed. ANB significantly decreased and SNB significantly increased in both groups after treatment, with no significant difference in the changes between the two groups. The mandibular length significantly increased in both groups, as measured by Co-Gn, Go-Gn and Ar-Gn; however, TB group did not show more mandibular growth than the RME group.

Significant differences in the changes in SE and NSAr were found between the two groups. SE and NSAr are related to the sagittal position of the mandible and reflect the position of the condyle [14]. The results showed that the increase in SE in the RME group was small, namely 1.10 mm. However, the increase in SE in the TB group was 2.23 mm, which was much larger than that in the RME group. Our results indicated that the condyle was inclined to significantly remodel backward after Twin-Block therapy. This finding is consistent with the finding of Yildirim et al. [15], who reported a backward and upward remodeling of the condyle after Twin-Block treatment. Moreover, the decrease in NSAr was much larger in the RME group, suggesting that the point Ar moved forward after RME therapy. This phenomenon might be explained by the obvious forward posture of the condyle after RME treatment [16]. 3D studies would provide more essential information.

The reason why RME therapy promotes mandibular growth in skeletal Class II patients has been controversial. The most accepted reason is that the increased space created by RME therapy relieves the block between the maxilla mandible, and enabled the mandible to naturally grow in three dimensions [17]. The second possible reason is the functional shift, created by occlusion disruption [18]. The boned acrylic and rapid displacement of the maxillary lateral segments might disrupt the occlusion and cause the patient to posture the mandible forward to a more comfortable position. Subsequently, with the remodeling and growth of the condyle, the initial postural change of the mandible becomes permanent [19, 20]. The third hypothesis suggested that the well-controlled vertical dimension in the RME group allows greater forward growth of the mandible, with less downward and clockwise rotation. Our results might support the second and the third reasons.

The facial profile improvements reflected by the Z Angle did not differ between the two groups. Both therapies proved to be useful in the correction of skeletal Class II malocclusion; however, RME therapy resulted in a similar sagittal mandibular growth with less clockwise rotation of the mandible in a shorter Phase I treatment period. This finding suggests that RME should be considered not only in patients with posterior crossbite, but also in hyperdivergent patients where an active growth response of the mandible is expected.

There are some limitations of this research. First, a control group is lacking. Mandibular growth could also be observed in a patient with no treatment. Therefore, a pure treatment effect on the mandibular growth following RME or Twin-Block therapy is lacking. Second, the lack of a CBCT examination resulted in debates about the relative contributions of real mandibular growth, mandibular functional shift and rotation of the mandible. Further studies using three-dimension evaluation might be helpful to provide more information.

## Conclusion

1. Both Phase I treatment with RME or Twin-Block followed by Phase II treatment of fixed appliance with four bicuspid extraction achieved significant increases in sagittal mandibular growth and obvious facial profile improvements in skeletal Class II patients.
2. RME treatment followed by fixed appliance was better for vertical control, because clockwise rotation of the mandible was avoided. In contrast, Twin-Block treatment followed by fixed appliance significantly increased the mandibular plane angle and caused an unfavorable clockwise rotation of the mandible, leading the mandible to grow downward and forward.
3. The phase I treatment period with RME therapy was shorter than that of Twin-Block therapy.

## Data Availability

The datasets used and/or analyzed during the current study are available from the corresponding author on reasonable request.

## Abbreviations

RME: Rapid maxillary expansion
TB: Twin Block
CVM: Cervical vertebral maturation
SD: Standard deviation
ICC: Intraclass correlation
